# Targeting of Pseudorabies Virus Structural Proteins to Axons Requires Association of the Viral Us9 Protein with Lipid Rafts

**DOI:** 10.1371/journal.ppat.1000065

**Published:** 2008-05-16

**Authors:** Mathew G. Lyman, Dusica Curanovic, Lynn W. Enquist

**Affiliations:** Department of Molecular Biology, Princeton University, Princeton, New Jersey, United States of America; Stanford University Medical School, United States of America

## Abstract

The pseudorabies virus (PRV) Us9 protein plays a central role in targeting viral capsids and glycoproteins to axons of dissociated sympathetic neurons. As a result, Us9 null mutants are defective in anterograde transmission of infection *in vivo*. However, it is unclear how Us9 promotes axonal sorting of so many viral proteins. It is known that the glycoproteins gB, gC, gD and gE are associated with lipid raft microdomains on the surface of infected swine kidney cells and monocytes, and are directed into the axon in a Us9-dependent manner. In this report, we determined that Us9 is associated with lipid rafts, and that this association is critical to Us9-mediated sorting of viral structural proteins. We used infected non-polarized and polarized PC12 cells, a rat pheochromocytoma cell line that acquires many of the characteristics of sympathetic neurons in the presence of nerve growth factor (NGF). In these cells, Us9 is highly enriched in detergent-resistant membranes (DRMs). Moreover, reducing the affinity of Us9 for lipid rafts inhibited anterograde transmission of infection from sympathetic neurons to epithelial cells *in vitro*. We conclude that association of Us9 with lipid rafts is key for efficient targeting of structural proteins to axons and, as a consequence, for directional spread of PRV from pre-synaptic to post-synaptic neurons and cells of the mammalian nervous system.

## Author Summary

Alpha herpesviruses are common mammalian pathogens (e.g. herpes simplex and chickenpox virus infect humans). These viruses enter and spread in and out of the mammalian nervous system, a defining hallmark of their lifecycle and potential pathogenesis. Neurons are polarized cells, and the movement of certain cellular proteins is highly restricted to the cell body, to dendrites, or to axons. Indeed, the axon harbors only a small subset of neuronal proteins. Thus, it is remarkable that these viruses efficiently gather and move their structural components from the cell body to the axon after infection of neurons. Previously, we identified a small viral membrane protein (known as Us9) that mediates the efficient targeting of virus particles to axons of infected neurons. We now report that Us9 must localize to “lipid raft” domains, specialized regions within cellular membranes to promote axonal targeting. Reducing the affinity of Us9 for lipid rafts dramatically reduces sorting of structural proteins to axons. This is the first report, to our knowledge, to implicate lipid rafts in targeting alpha herpesvirus structural proteins to axons, an essential step for spread of infection in the mammalian nervous system.

## Introduction

Neurons are highly polarized cells with distinct biochemical and functional properties particular to the cell body (soma), dendrites, and the axon. Soluble, cytosolic proteins likely move into axons and dendrites by bulk flow, while membrane proteins are highly restricted to the somatodendritic membrane, the axonal membrane, or within vesicles [1]. Sorting of membrane proteins to the somatodendritic or axonal compartments of neurons is similar to the sorting mechanism of membrane proteins either to the basolateral or to the apical membranes of epithelial cells [2]. Both cell types share a common component for compartmentalization of their membrane milieu: lipid raft microdomains. Rafts often are defined biochemically as detergent-resistant membranes (DRMs) or detergent-insoluble glycolipid complexes (DIGs) [3]–[5]. These protein-lipid complexes are highly enriched in cholesterol and sphingolipids, and float freely within the liquid-disordered state of the lipid bilayer. Furthermore, they are small and dynamic, often ordering themselves into larger, more stable rafts through protein-protein and protein-lipid interactions [6],[7].

Lipid rafts/DRMs play a key role in the axonal sorting of membrane proteins during neuronal maturation, including axonal growth and guidance [8]. Ledesma et al. showed that two axonally-targeted membrane proteins, Thy-1 and influenza virus hemagglutinin (HA), interact with sphingolipid-cholesterol rafts in rat hippocampal neurons [9]. These microdomains normally were resistant to detergent treatment at 4°C. However, reducing the levels of cholesterol and sphingolipids resulted in the detergent-solubility of both proteins, as well as aberrant sorting into axons [9]. The increased synthesis of sphingomyelin during neuronal maturation was critical for the formation of protein-lipid complexes, an essential step for the targeting of randomly distributed GM1 ganglioside in immature neurons to the axon of fully mature neurons [4].

Alpha herpesviruses (e.g. herpes simplex virus (HSV) and pseudorabies virus (PRV)) replicate and traffic within polarized neurons, a strategy conducive to their lifestyle in the host peripheral nervous system (PNS). Infection begins with virion entry at mucosal surfaces and spread of infection between cells of the mucosal epithelium. The PNS is infected through axon termini innervating this region, and subsequent trafficking of capsids to the cell body. It is here that a reactivatable, latent infection is established that persists for the life of the host [10]. A well known, but poorly understood observation, is that upon reactivation from the latent infection, α-herpesviruses rarely enter the central nervous system despite having what seems to be two rather similar choices: cross one synapse and infect the central nervous system (rare) or traffic back down the axon to the initial peripheral site of infection (very common). Inherent in this choice is the fact that viral proteins must be targeted to axons, a highly specialized neuronal compartment restricted to only a subset of neuronal proteins. The primary problem is to identify the mechanisms that gather and sort the many viral structural proteins to this compartment.

The Us9 gene product, a small, tail-anchored type II membrane protein, has been of interest in understanding the mechanisms of anterograde transport of alpha herpesviruses in the mammalian nervous system [11]–[14]. PRV Us9 directs viral membrane proteins and capsids to axons of infected neurons [15],[16], an absolute requirement for anterograde neuron-to-cell and neuron-to-neuron spread of the virus [11],[17]. Our current data demonstrate that in the absence of PRV Us9, axons do not contain vesicles with viral glycoproteins or mature virus particles [15]. However, the mechanism by which this occurs has not been elucidated.

Favoreel et al. reported that PRV glycoproteins gB, gC, gD, and gE were associated with lipid rafts on the surface of infected swine kidney cells and monocytes [18]. PRV Us9 is essential for the trafficking of these glycoproteins into the axon of dissociated superior cervical ganglia (SCG) neurons [16]. These observations, in conjunction with reports that raft association is critical for axonal targeting of certain neuronal membrane proteins [4],[9],[19] led us to investigate whether PRV Us9 was associated with detergent-insoluble lipid rafts.

## Materials and Methods

### Virus Strains

All PRV strains were propagated on porcine kidney (PK15) cells at a low multiplicity of infection (MOI) for 48 hours and then collected by scraping cells into the conditioned medium as described previously [15]. The wild-type PRV strain Becker and its derivatives, PRV 99, PRV 160, and PRV 162 were described previously [11], [20]–[22]. PRV 99 is deleted for the sequence encoding both gE and gI. PRV 160 (Us9-null) contains a nonsense stop mutation at position 4 in the Us9 open reading frame. PRV 162 encodes a mutant Us9 protein in which the nucleotide sequence encoding amino acids 46 to 55 have been removed (i.e. the acidic cluster region).

To construct PRV 322, the wild-type transmembrane domain of PRV Us9 was replaced with that of the transferrin receptor (TfR) transmembrane domain [23],[24]. This was performed by the SOEing PCR method [25] using pML47 as the template. This plasmid contains the *SpH*I/*Mlu*I region of the Becker *Bam*HI 7 fragment cloned into pBluescript SK(+).The upstream fragment was amplified using the upstream-forward primer 5′ GCATGCTCTCGCCGGTGT 3′ and the downstream-reverse primer 5′ aaatccaatcaagaaaaagacgatcacag aatagtcccatagcagatacttccactaca GCGGCGGCGTCTCCGGCG 3′. The downstream fragment was amplified using the upstream-forward primer 5′tattgctgtgatcgtctttttcttgattggatttatgattggctacttgggctattgtCGGCACGTGTAGCGAGCGGGT3′ and the downstream-reverse primer 5′ ACGCGTAGCACCACTCGG 3′. Upper case letters represent PRV sequences whereas lower case letters represent sequences introduced to synthesize the TfR TMD domain. Both the upstream and downstream fragments were gel-purified, and ligated together using the upstream-forward primer 5′ GCATGCTCTCGCCGGTGT 3′ and the downstream-reverse primer 5′ ACGCGTAGCACCACTCGG 3′. The SOEing product was cloned directly into the pCR-Blunt II-TOPO vector (TOPO Cloning Kit, Invitrogen, Carlsbad, CA) and sequenced to verify that no extraneous mutations were introduced during PCR. This plasmid was designated pML55. We then digested pML55 with the restriction enzymes *Bsr*GI and *Mlu*I to release a ∼1.0 kb fragment that contained the Us9 gene with the TfR transmembrane domain. This fragment was used to replace the *Bsr*GI/*Mlu*I fragment of pML68, a pT7Blue vector backbone containing a *Sal*I/*SpH*I fragment spanning a large portion of the unique-short region (a splice product of the *Sal*I/*Mlu*I fragment of pPH 2 and the *Mlu*I/*SpH*I fragment from pGS166). It includes a portion of the gD gene, gI, gE, Us9, and virtually the entire Us2 gene. This construct (designated pML73) was digested with *Eco*RI/*Hind*III and cotransfected with purified PRV 165 genomic DNA [26] into PK15 cells. Plaques not expressing EGFP were identified using a Nikon Eclipse TE300 inverted epifluorescence microscope and then subjected to three rounds of plaque purification. This recombinant virus was named PRV 319. Initial characterization of PRV 319 revealed that Us9-TfR unexpectedly formed a dimer inside infected PK15 and PC12 cells (unlike the monomeric form of wild-type Us9). We discovered that two cysteine residues at the N- and C-terminus of the TfR transmembrane domain were responsible for disulfide-bonded dimer formation and protein acylation [27]. We deleted these codons from pML55 using the Stratagene Quickchange II site-directed mutagenesis kit (Stratagene, La Jolla, CA). This construct, denoted pML86, was digested with the restriction enzymes *Bsr*GI and *Mlu*I and the 1 kb fragment was cloned into the pML68 shuttle vector. This construct (designated pML88) was digested with *Eco*RI/*Hind*III and cotransfected with purified PRV 165 genomic DNA [26] into PK15 cells. Non-green plaques were identified and purified as described above. This recombinant virus was designated PRV 322. The presence of the Us9-TfR chimeric protein, as well as the presence of gE and Us2 (genes directly upstream and downstream of Us9, respectively) were verified by western blot of infected cell lysates.

### Antibodies

Antibodies used in this report include: polyclonal rabbit antiserum recognizing Us9 ([28]; used 1∶500 by WB and 1∶200 by IF), the gE cytoplasmic domain ([29]; used 1∶1000 by WB), and PRV antigens (Rb134 [21],[22]; used 1∶10,000 by IF), polyclonal goat antiserum recognizing gB, gC, Us2, and UL34 ([22],[30]; all used 1∶1000 by WB), and mouse monoclonal antibody recognizing transferrin receptor (Zymed, San Francisco, CA). The rabbit polyclonal gH antiserum was a kind gift from Thomas Mettenleiter [31]. All secondary Alexa fluorophores (used at 1∶500) were purchased from Molecular Probes.

### PC12 Cultures

A detailed protocol for culturing and differentiating PC12 cells prior to infection with PRV has been published previously [32]. Briefly, we coated the surface of a 150 mm dish with rat tail collagen (type 1) at a concentration of 5 µg/cm^2^ in 0.02 N ascetic acid for 1 hour. The dish was washed gently three times with sterile water, and 20 ml of complete growth medium (85% RPMI, 10% horse serum, 5% fetal calf serum) was added to the plate. A 100 mm dish with undifferentiated PC12 cells (∼80% confluency) was split 1∶5, and triturated extensively with a sterile 5 ml plunger and 22-gauge needle to dissociate cell clumps. After trituration, 1 ml of cell suspension was added to the 150 mm dish, and cells were allowed to attach overnight in a 37°C incubator. To produce a culture of differentiated PC12 cells, growth medium was replaced with differentiation medium (RPMI, 1% horse serum, nerve growth factor (100 ng/ml)) when cells became ∼30% confluent. Differentiation medium was replaced every other day for 12 days, at which time an extensive network of neurites was visible. Undifferentiated PC12 cells were propagated in growth medium to ∼70% confluency prior to infection with PRV.

### Live-cell imaging of infected PC12 cells

Live imaging of viral capsids was performed using a Leica SP5 with an HCX Plan Apochromat 63×1.3 NA glycerin objective. Approximately 15 optical sections were acquired in 0.5-µm steps through the plane of the neurites and cell body. Each frame of the movie is a 2D projection of one stack of images. Prior to imaging, 1 M HEPES was added to the RPMI medium to a final concentration of 25 mM. PC12 cells were cultured on MatTek Corp. glass-bottomed dishes (http://www.glass-bottom-dishes.com/). The dish was warmed to 37°C employing a DH40i Micro-incubation System (Warner Instrument Corp.) run at constant voltage (∼5.5 volts). A 488 nm laser line was used for GFP excitation (10% intensity), with emissions collected from 495 to 553 nm. Images were acquired employing a 1.5 airy unit detector pinhole and scanning at 700 Hertz. All figures were assembled in Adobe Photoshop 7.0.1. Movies were created using ImageJ 1.32j software (National Institutes of Health).

### Lipid raft flotation assay

Flotation of lipid rafts by Optiprep™sucrose gradient is well documented in the literature [33]–[35], though subtle differences exist between protocols. We followed those described previously for PRV-infected SK cells [18] and uninfected PC12 cells [36]. Undifferentiated and differentiated PC12 cells were cultured in a 150 mm dish as described above (∼10^7^ cells). Cells were infected at a high multiplicity of infection (MOI = 10) with PRV Becker, PRV 99, PRV 162, or PRV 322. At 12 hours post-infection (hpi), cells were collected in a 50 ml conical tube, and washed twice with cold RPMI medium by brief centrifugation at 3,000 rpm. Cells were lysed with 1 ml of lysis buffer consisting of 1% TX-100 in TNE buffer (25 mM Tris HCl [pH 6.8], 150 mM NaCl, 5 mM EDTA), protease inhibitor cocktail (Roche Diagnostics GmbH, Mannheim, Germany), and 5 mM iodoacetamide. The lysate was homogenized by being passed 15 times through an 18-gauge needle, and then allowed to rock for 30 minutes at 4°C. At the end of the rocking period, the sample was again homogenized briefly, and then mixed with 2 ml of ice-cold 60% Optiprep™ density gradient medium (Sigma-Aldrich, St. Louis, MO). The entire 3 ml mixture was placed at the bottom of a Beckman SW41 ultracentrifuge tube (Beckman, Munich, Germany) and subsequently overlaid with 5 ml of ice-cold 30% Optiprep in TNE and 4 ml of ice-cold 5% Optiprep in TNE. Samples were centrifuged at 34,200 rpm (200,000×g) at 4°C for 20 hours. Twelve fractions were collected from the top to the bottom of the tube (1 ml each), and mixed 1∶1 with 2× Laemmli sample buffer. Samples 3–10 were electrophoresed on a 12% SDS-PAGE gel.

### Single-step growth curve

PK15 cells were grown to confluency in 60 mm dishes (∼3×10^6^ cells/dish), and infected with the indicated viruses at an MOI of 10. Following a one-hour adsorption period at 37°C, the cells were rinsed once with PBS and incubated at room temperature for three minutes with 3 ml of citrate buffer (40 mM sodium citrate, 10 mM KCl, 135 mM NaCl, pH 3.0) to inactivate unabsorbed virus. The cells were rinsed three times with PBS, 2 ml of fresh medium were added, and plates were returned to the incubator. At various times post-infection, cells were scraped into the medium and frozen in two aliquots. Aliquots were freeze-thawed three times (−80°C/+37°C) and titered in duplicate on PK15 cell monolayers.

### Virion purification

Virions were purified as described previously [28]. Briefly, three confluent 150-mm-diameter dishes of PK15 cells were infected with virus at a MOI of 10. At 16 hpi, medium was collected and centrifuged at 3,000 rpm to remove cellular debris. The clarified supernatant was layered on a 7 ml 30% sucrose-PBS cushion (w/v) in two Beckman SW28 centrifuge tubes. The tubes were then centrifuged in an SW28 rotor at 23,000 rpm for 3 hours. The sucrose cushion was removed, and the virion pellet was resuspended in 1 ml of PBS by 10 one-second pulses in a bath sonicator and gentle pipetting. Virions were then centrifuged through a 1 ml 30% sucrose cushion at 28,000 rpm for 90 minutes in an SW55ti rotor. The pelleted virions were resuspended in 100 µl PBS, and subsequently mixed with Laemmli sample buffer for analysis by SDS-PAGE.

### Western Blot Analysis

To analyze steady-state viral protein expression, PK15 cells (∼3.0×10^6^) were either mock infected or infected at a high MOI with Becker or PRV 322. At 6 hpi, medium was removed and infected cells were scraped into 330 µl of PBS and 170 µl of 3× Laemmli sample buffer. Samples were homogenized with an insulin syringe, boiled for 2 minutes, and subjected to sodium dodecyl sulfate polyacrylamide gel electrophoresis (SDS-PAGE). Preparation of lipid raft fractions and purified virions for SDS-PAGE is described above. All gels were transferred to an Immobilon-P membrane using a semidry transfer apparatus following the manufacturer's instructions (Labconco, Kansas City, MO). Following transfer, membranes were immediately put into blocking solution (2% bovine serum albumin in TBS (50 mM Tris, 200 mM NaCl) (w/v), 0.1% Tween-20 (v/v)) and incubated for 15 minutes at RT. Primary antibody was diluted in 2% BSA-TBS-Tween solution, and allowed to rock for 30 minutes with the membrane. GM1 was detected using biotinylated cholera toxin B subunit (1 µg/ml, Sigma-Aldrich, St. Louis, MO) and a HRP-streptavidin conjugate (1∶1000, Pierce, Rockford, IL). Following the 30 minute incubation, the membrane was washed three times with TBS-Tween, and placed in HRP-conjugated secondary antibody (1∶10,000, KPL, Gaithersburg, Maryland) diluted in 2% BSA-TBS-Tween for 30 minutes. The membrane was then washed as previously described, and proteins were visualized with the ECL Plus Western blotting detection system (GE Healthcare).

### Imaging of transfected and infected PK15 cells

PK15 cells were grown on glass coverslips (∼20% confluence) and transfected with mammalian expression vectors encoding GFP (pEGFP-N1), Us9-GFP (pBB14), and Us9-TfR-GFP (pML92) using Lipofectamine 2000 as directed by the manufacturer's instructions (Invitrogen, Carlsbad, CA). At 24 hours post-transfection, cells were washed with PBS and fixed with 4% paraformaldehyde in PBS for 10 min. After fixation, cells were washed three times with PBS, and stained with Hoechst 33342 (Invitrogen, Carlsbad, CA). Samples were then mounted on a glass slide using Aqua poly/mount (Polysciences, Warrington, PA) and allowed to air dry for 24 hours prior to imaging. Direct fluorescence was visualized using an inverted epifluorescence microscope and the appropriate excitation and emission filters. For indirect immunofluorescence experiments, PK15 cells were grown to 30% confluence on glass coverslips and infected with Becker, PRV 160, and PRV 322. At 6 hpi, cells were washed three times with phosphate-buffered saline (PBS), then fixed with 4% paraformaldehyde in PBS for 10 min. After fixation, cells were washed three times with PBS, and permeabilized for 3–5 minutes with 0.5% Triton X-100 in PBS. After permeabilization, Us9 antiserum was diluted 1∶200 in wash buffer (PBS, 3% BSA, 0.5% saponin) and added to cells for 1 hour. Primary antibody was then removed, and the sample washed three times. Next, secondary antibodies were added to the sample and incubated for 1 hour. Secondary antibody was then removed and the sample was washed an additional three times. Samples were mounted on a glass slide using Aqua poly/mount and allowed to air dry for 24 hours prior to imaging. Optical sections were acquired using a Leica SP5 confocal microscope with a 63×/1.3 NA oil objective.

### Neuronal cultures

Detailed protocols for dissecting and culturing PNS neurons from rat embryos have been published previously [32]. Briefly, sympathetic neurons from the superior cervical ganglia (SCG) were dissected from rat embryos at embryonic day 15.5 to 16.5 (Sprague-Dawley rats, Hilltop Labs, Inc., Scottdale, PA) and incubated in 250 µg/ml of trypsin (Worthington Biochemicals) for 10 min. Trypsin inhibitor (1 mg/ml; Sigma Aldrich) was added to neutralize the trypsin for 5 min and then removed and replaced with neuron culture medium (described below). Prior to plating, the ganglia were triturated using a fire-polished Pasteur pipette and then plated in the S compartments of the Teflon ring. The Teflon ring was placed within a 35-mm plastic tissue culture dish coated with 500 µg/ml of poly-DL-ornithine (Sigma Aldrich) diluted in borate buffer and 10 µg/ml of natural mouse laminin (Invitrogen). The neuron culture medium is serum free and consists of Dulbecco's modified Eagle medium (Invitrogen) and Ham's F12 (Invitrogen) in a 1∶1 ratio. The serum-free medium was further supplemented with 10 mg/ml of bovine serum albumin (Sigma Aldrich), 4.6 mg/ml glucose (J. T. Baker), 100 µg/ml of holotransferrin (Sigma Aldrich), 16 µg/ml of putrescine (Sigma Aldrich), 10 µg/ml of insulin (Sigma Aldrich), 2 mM of L-glutamine (Invitrogen), 50 µg/ml or U of penicillin-streptomycin (Invitrogen), 30 nM of selenium (Sigma Aldrich), 20 nM of progesterone (Sigma Aldrich), and 100 ng/ml of nerve growth factor 2.5S (Invitrogen). Two days after plating, the neuronal cultures were treated with 1 µM of antimitotic drug cytosine ß-D-arabinofuranoside (AraC; Sigma Aldrich) to eliminate any nonneuronal cells. The neuron culture medium was replaced every 3 to 4 days, and cultures were maintained in a humidified, CO_2_-regulated, 37°C incubator. All experimental protocols related to animal use were approved by the Institutional Animal Care and Use Committee of the Princeton University Research Board under protocol number 1691 and are in accordance with the regulations of the American Association for Accreditation of Laboratory Animal Care and those in the Animal Welfare Act (public law 99-198).

### Trichamber culture system

Protocols for assembling the trichamber system have been described previously [17],[37]. Tools and reagents, including the Teflon rings (Tyler Research, Alberta, Canada) and the silicone grease-loaded syringe (Dow Corning), were sterilized by autoclaving prior to assembly. Tissue culture dishes (35 mm) were coated with 500 µg/ml of poly-DL-ornithine (Sigma Aldrich) followed by 10 µg/ml of natural mouse laminin (Invitrogen), and then washed and dried; the bottom surface of each dish was etched with a pin rake, creating a series of 16 evenly spaced grooves. We used a silicone grease-loaded syringe attached to an 18-gauge truncated hypodermic needle to apply a thin, continuous strip of silicone grease over the entire bottom surface of the Teflon ring. Next, a 50-µl drop of neuron medium containing 1% methocellulose (serum free) was placed in the center of each tissue culture dish covering the etched grooves. This step prevents the seal from being entirely devoid of moisture, which is needed for axon penetration and growth between the grooves. Finally, the silicone grease-coated ring was gently seated on the tissue culture dish or the surface of the 35-mm dish such that the etched grooves spanned all three compartments, forming a watertight seal between compartments. Neuron medium was then placed in all three compartments immediately after the chamber was assembled. Once the SCG neurons were dissected and dissociated, approximately one half of a single ganglion was plated into the S chamber. Neuron cultures were then maintained according to the protocols for culturing neurons reported above.

### Assaying neuron-to-cell spread of infection

Neurons were cultured for approximately 2 weeks in the trichamber system (on 35 mm tissue culture dishes) with frequent medium changes. After 2 weeks, axon penetration into the M and N compartments was assessed visually and only cultures with comparable axon densities were used for experiments. After axons penetrated the N compartment, nonneuronal epithelial cells (PK15 cells) permissive for PRV infection were plated in the N compartment. The neuron medium in the N compartment was supplemented with 1% fetal bovine serum and the cells were allowed to attach and expand for 24 h prior to any experiment. Once the target cells in the N compartment were plated, neuron medium containing 1% methocel was placed in the M compartment. After 30 min, the neuronal cell bodies in the S compartment were infected with virus diluted in neuron medium (approximately 10^5^ PFU). After 1 h, the viral inoculum was removed and replaced with neuron medium. The chambers were then incubated in a humidified 37°C incubator for 24 hours. Both intracellular and extracellular virions in the S and N compartments were carefully harvested by scraping the bottom of the dish with the pointed end of a gel-loading tip. The cells and medium were then pooled and freeze-thawed, and titers were determined.

### Indirect Immunofluorescence in the Trichamber culture system

Trichambers were assembled on UV-sterilized Aclar strips. Neurons were cultured and infected as described above. At 16 hpi, all compartments were washed twice with PBS containing 3% BSA (PBS/BSA), chambers gently lifted, and silicone grease scraped off the Aclar strips. Samples were then fixed with 4% paraformaldehyde in PBS for 10 minutes. Fixative was washed away with three PBS/BSA rinses, after which the samples were permeabilized using a solution of 0.5% saponin and 3% BSA in PBS (PBS/BSA/SAP). Incubations with primary and secondary antibodies were performed for one hour in PBS/BSA/SAP. Following two rinses with PBS/BSA/SAP and one rinse with distilled water, the samples were mounted on glass slides using Aqua poly/mount (Polysciences). Images were collected on a Perkin-Elmer RS3 spinning disk confocal microscope using a 40×1.3 NA oil objective. Z-stacks obtained in 1-micron steps were collapsed and analyzed in Perkin-Elmer ImageView software.

## Results

The characterization of lipid rafts has largely been based on their resistance to detergent solubilization at 4°C [3]. This approach continues to be a useful tool to assess the affinity a protein has for lipid rafts when formed at physiologic temperatures [38],[39]. Analyses performed on changes in DRM partitioning (due to a physiological stimulus) have provided the foundation for extensive work on the role of lipid rafts in signal transduction [40]–[42], membrane trafficking [43],[44], and pathogenesis [45]. Accordingly, we investigated the affinity of Us9 for DRMs, as well as several viral glycoproteins whose axonal localization is dependent on Us9 [16].

**Figure 1:**
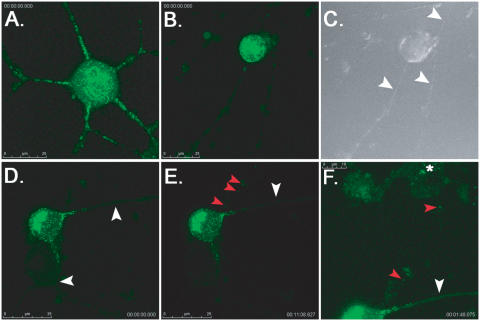
Live-cell imaging of GFP-tagged capsid viruses in differentiated PC12 cells. Cells were grown on glass-bottom MatTek dishes coated with poly-DL-ornithine and natural mouse laminin prior to imaging on a Leica SP5 confocal microscope. Each frame of the movie, a 2D projection representing a stack of 15 images that are 0.5 µm apart, contains a scale bar (in microns) and timestamp from the movie sequence. (A) A plate of differentiated PC12 cells was infected with PRV GS443 (green capsid) at a low MOI (0.1) and imaged at 12 hours post-infection (hpi). Numerous green capsid puncta were observed in neurites moving in the anterograde direction, i.e. away from the cell body (see Video S1 in supplemental material). This cell was chosen because it had several axon projections to emphasize the sorting phenotypes. Most differentiated PC12 cells have fewer axon projections (1–3). (B) Differentiated PC12 cells were infected with PRV 368 (green capsid, Us9-null) at low MOI and imaged at 12 hpi. The cell body shows robust green fluorescence, but virus capsids were not observed in neurites emanating from the cell body (see Video S2). (C) DIC image showing three neurites extending from the PRV 368-infected cell body (highlighted by the white arrowheads). (D) A differentiated PC12 infected with PRV 368 for 12 hours. No capsids are observed moving in the anterograde direction in two neurites emanating from the cell body (white arrowheads, see Video S3). Note that capsids are not present beyond the proximal segment of the axon. (E and F) Though no green capsid puncta are moving in the anterograde direction (white arrowheads), capsids can be observed moving in a transneuronal, retrograde manner from the infected PC12 cell (red arrowheads, see Video S3) to an uninfected cell above it (panel F, see Video S4). Despite an abundance of moving capsid puncta within the cell body, no other egress events are visible. The brightness in panel F has been increased to better visualize green capsid puncta moving into the uninfected cell body (highlighted by red arrowheads). A white asterisk denotes the accumulation of capsid puncta in the cell body of the uninfected cell.

It is difficult to culture a sufficient number of primary rat neurons to perform large biochemical analyses. Therefore, we used PC12 cells, a widely used rat pheochromocytoma cell line that responds to nerve growth factor (NGF) and acquires many of the characteristics of sympathetic neurons [46]. Differentiated PC12 undergo polarized protein sorting, and cell bodies stain for nonphosphorylated neurofilament H (a somatodendritic marker) while axons stain exclusively for phosphorylated neurofilament H (an axonal marker) [32]. This is consistent with mature, sympathetic SCG neurons [47]. It has been reported that PC12 cell are susceptible and permissive to PRV infection [48], and that a PRV GFP-VP22 fusion protein moves inside neurites with fast axonal kinetics [49]. However, it was unclear whether the Us9-null phenotype in SCG neurons, i.e. a complete block to axonal sorting of viral structural proteins [15],[16], could be recapitulated in this neuron-like cell line (a critical experiment to ensure that PC12 cells could be used to study Us9 biology). We recently reported that in the absence of Us9, GFP-tagged capsids were unable to sort into axons of live SCG neurons [15]. Therefore, we utilized a similar live-cell imaging approach to examine the axonal sorting of GFP-tagged capsids in differentiated PC12 cells. Cells were infected with PRV GS443, a recombinant PRV strain that expresses GFP fused to VP26, a capsid protein [50]. After 12 h, capsid puncta were readily observed trafficking in the anterograde direction within neurites of PC12 cells (n = 20) (Figure 1A, Video S1). Importantly, when differentiated PC12 cells were infected with PRV 368, a GFP-tagged capsid mutant deleted for Us9, no green puncta were observed moving in the anterograde direction (Figure 1B and 1C, Video S2). These findings were consistent with our Us9 studies in dissociated SCG neurons [15]. Interestingly, we also observed the retrograde trafficking of capsid puncta from cells infected with PRV 368 to uninfected, neighboring cells (in the absence of any anterograde sorting of virus particles in the same field of view) (Figure 1D–1F, Videos S3 and S4). This had been described previously in transneuronal spread studies on Us9 mutants in the rat visual system [11],[26], but had not been observed in tissue culture cells. It is noteworthy that we did not visualize “random” egress of GFP-tagged capsids from infected cell bodies. Capsids either sorted into axons (in the presence of Us9), or to sites of synaptic contact with other axons (transneuronal, retrograde transport). The import of this observation is unclear at present, but may suggest that alpha herpesviruses undergo directed egress from neuronal cell bodies. Overall, our findings suggest that differentiated PC12 cells recapitulate the Us9 sorting phenotypes previously observed in primary sympathetic neurons, and are an efficacious cell line to study Us9 biology (specifically that of Us9 and lipid rafts).

**Figure 2:**
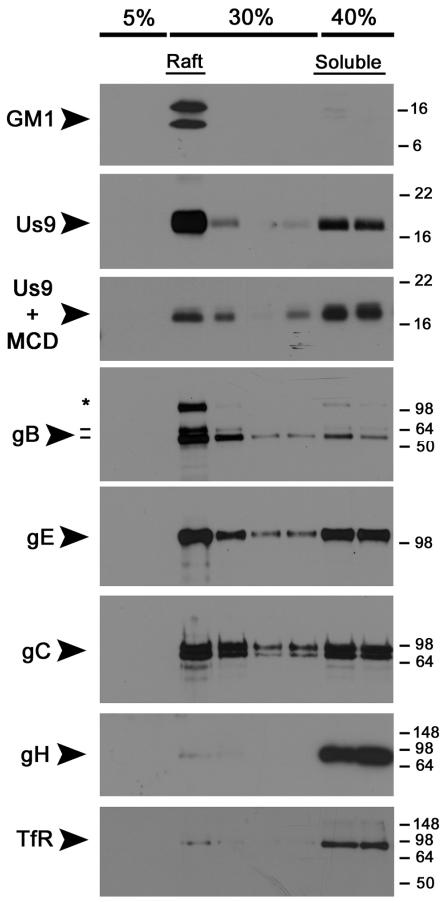
PRV Us9 and gB are highly-enriched in DRMs of non-polarized PC12 cells, whereas gH is not. Non-differentiated PC12 Cells were infected with PRV Becker for 12 hours, and then lysed with cold 1% Triton X-100. Lysates were separated on a discontinuous Optiprep™density gradient, and 10 fractions were collected from the top to the bottom of the tube (1 ml each). Samples 2–9 were subjected to SDS-PAGE, and Western blotting analysis was performed using biotinylated cholera toxin B subunit (for GM1) and antibodies to PRV Us9, gB, gE, gC, gH and transferrin receptor (TfR). To test the effect of cholesterol depletion on Us9 association with DRMs, infected PC12 cells were incubated with 20 mM methyl-cyclodextrin (MCD) at 37°C for 45 minutes prior to lysis with cold detergent. The preprocessed form of gB (*) is labeled along with the 69 kDa and 58 kDa (−) subunits.

Therefore, we compared the raft profiles of viral membrane proteins after infection of non-polarized and polarized PC12 cells. Undifferentiated cells (∼10^7^) were infected with wild-type Becker for 12 hours, solubilized with 1% TX-100, and subjected to a well-described “raft flotation” assay [33]–[35]. Membrane proteins that were solubilized by TX-100 remained at the bottom of the Opti-Prep gradient (40%), whereas proteins in DRMs floated to the 5%–30% interface. We used the prototypic raft and non-raft markers, GM1 ganglioside and transferrin receptor (TfR), as positive and negative controls [51]. Us9 was highly enriched in the raft fraction as compared to the soluble population (Figure 2). To test whether the flotation of Us9 was cholesterol dependent, we treated cells with methyl-cyclodextrin (MCD) prior to solubilization with detergent. MCD depletes cholesterol from cellular membranes, and therefore disrupts the structure of lipid raft microdomains [52],[53]. A 45-minute exposure of 20 mM MCD to Becker-infected cells dramatically decreased the amount of Us9 floating with the raft fraction. We found the viral glycoprotein gB to have a strong affinity for DRMs while gE and gC where not enriched in either the raft or soluble fractions. These findings were consistent with similar experiments performed in non-polarized swine kidney (SK) cells [18]. Surprisingly, PRV gH was completely solubilized by TX-100 treatment, as was transferrin receptor (the negative control). Overall, Us9 and gB have a strong affinity for DRMs in undifferentiated PC12 cells. Both gE and gC were present in the raft and soluble fractions in a ∼1∶1 ratio, whereas gH was completely soluble.

**Figure 3:**
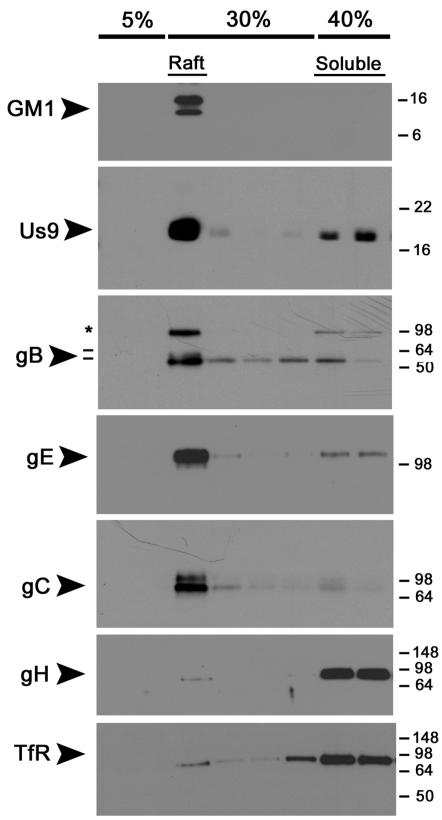
Differentiation of PC12 cells with nerve growth factor (NGF) increases the association of PRV gE and gC with DRMs, but gH remains soluble. PC12 cells were differentiated in low serum conditions in the presence of NGF for 12 days. Cells were infected with PRV Becker for 12 hours, and then lysed with cold 1% Triton X-100. Lysates were separated on a discontinuous Optiprep™ density gradient, and analyzed by SDS-PAGE. Western blotting was performed using biotinylated cholera toxin B subunit (for GM1) and antibodies to PRV Us9, gB, gE, gC, gH and transferrin receptor (TfR). The preprocessed form of gB (*) is labeled along with the 69 kDa and 58 kDa (−) subunits.

To determine the effect of cell polarization on DRM partitioning, we cultured PC12 cells in low serum conditions in the presence of nerve growth factor for 12 days. This treatment allows for extensive neurite outgrowth from the cell bodies, as well as separation of somatodendritic and axonal marker proteins [32]. We infected cells with PRV Becker for 12 hours, and subjected lysates to raft flotation analysis as performed previously (Figure 3). Us9 and gB were again strongly associated with the raft fraction of the gradient, and floated with GM1. Both gE and gC were highly enriched in the DRM fraction of PC12 cells upon differentiation with NGF. This finding is consistent with the notion that polarization of neurons strongly impacts the affinity and targeting of certain membrane proteins to lipid rafts/DRMs [4]. gH was completely solubilized by TX-100 even in differentiated PC12 cells, and remained in the soluble fraction with TfR. These data suggest that Us9 and gB have a high affinity for DRMs in undifferentiated and differentiated PC12 cells, whereas gC and gE increase their association with DRMs as cells polarize and mature.

**Figure 4:**
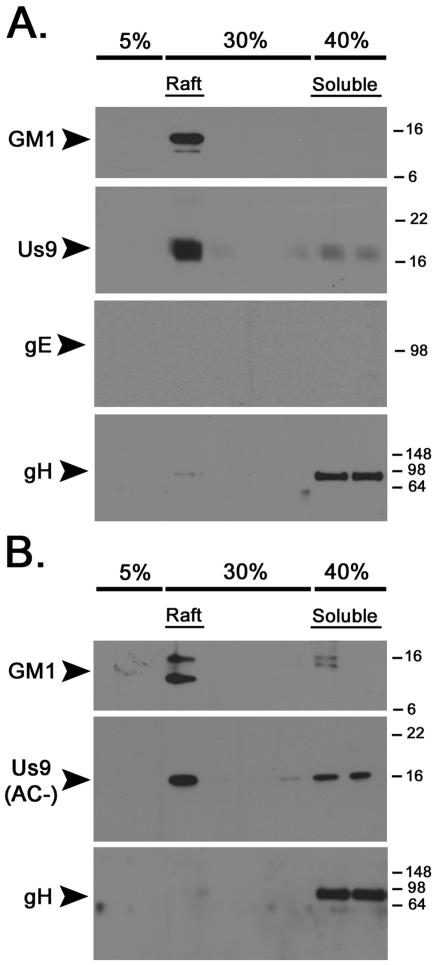
The raft association of Us9 is not dependent on the presence of gE or the Us9 acidic cluster motif. Non-differentiated PC12 Cells were infected with PRV 99 (A) or PRV 162 (B) for 12 hours. Cells were subsequently lysed with cold 1% Triton X-100, and separated on a discontinuous Optiprep™ density gradient. Samples were subjected to SDS-PAGE, and the presence of GM1, Us9, gE, and gH was assessed by Western blot analysis.

The efficient targeting of viral structural components to the axon of infected cells is dependent on both the Us9 and gE gene products [15],[16],[54]. Deletion of either gene results in the reduction of viral capsids and enveloped proteins in the axon, and subsequent reduction of anterograde spread of infection *in vitro* and *in vivo* [11],[17],[55]. Upon discovering that both Us9 and gE were present in the DRMs of infected PC12 cells, we tested whether deleting the gE/gI complex affected the ability of Us9 to target to rafts, thereby impacting its ability to function properly in anterograde transport. PC12 cells were infected with PRV 99, a mutant deleted for the gE and gI genes, and subjected to raft flotation analysis. Deletion of gE/gI had no affect on the ability of Us9 to associate with DRMs (Figure 4A), nor did the absence of gB (data not shown). These data again support the notion that Us9 has an intrinsic affinity for DRMs and is not influenced by cell polarity or the presence of two major viral DRM components. It is noteworthy that we found no aberrant targeting of gB, gC, or gE to DRMs in a Us9-null mutant (data not shown).

It is well documented that certain proteins become raft associated upon their phosphorylation during signal transduction [56]–[58]. Us9 contains a conserved acid cluster (AC) region with two serines that are phosphorylated [26], as well as a di-tyrosine motif critical for anterograde, transynaptic spread [26]. To examine whether inhibiting Us9 phosphorylation precluded Us9 association with DRMs, we infected PC12 cells with PRV 162, a mutant that expresses an altered Us9 protein lacking the acidic cluster region. DRMs were prepared from infected cells as performed previously (Figure 4B). Us9 was still enriched in the DRM fraction of the gradient despite the absence of Us9 phosphorylation (note the narrowness of the Us9 band compared to wild-type Us9 in panel A). Taken together, these data suggest that Us9 is highly enriched in DRMs, and its affinity for this lipid microdomain is not dependent on its phosphorylation state.

Both the influenza virus neuraminidase (NA) and haemagglutinin (HA) proteins (both type II membrane proteins) are highly enriched in DRMs/lipid rafts, and this association is critical for the apical sorting of these proteins in polarized MDCK cells [59]–[61]. Importantly, the transmembrane domain (TMD) of these proteins provided the determinants for apical sorting and raft association [59], [62]–[64]. This was demonstrated by swapping the TMD of transferrin receptor (a type II, non-raft associated membrane protein) for the TMD of neuraminidase [63]. Transferrin receptor was normally sorted to the basolateral membrane in polarized MDCK cells, and was efficiently solubilized by 1% TX-100. By contrast, transferrin receptor with the NA TMD was targeted to the apical cell surface and was largely insoluble to treatment with TX-100 [59]. In a reciprocal experiment, the neuraminidase TMD was replaced with the transferrin receptor TMD [62]. This chimeric protein was greatly reduced in lipid raft association, and a virus expressing this protein showed a defect of particle release from the apical cell surface.

**Figure 5:**
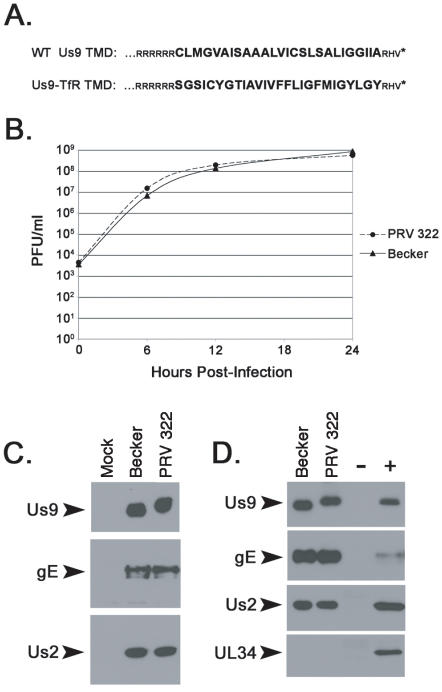
Characterization of PRV 322 (Us9-TfR). (A) The amino acid alignment of the C-terminal portion of wild-type Us9 and Us9 with the transferrin receptor transmembrane domain (Us9-TfR). The transmembrane domain sequences are denoted in large, bold letters. (B) Single step growth analysis on Becker and PRV 322 in PK15 cells. (C) The steady-state expression of Us9, gE, and Us2 in PK15 cells infected with Becker and PRV 322 (6 hpi). (D) Incorporation of Us9, Us9-TfR, gE, and Us2 into purified mature virions, but not UL34. Mock and Becker infected lysates serve as the negative (−) and positive (+) controls, respectively.

We employed a similar approach with PRV Us9 to test whether its transmembrane domain provided the determinants for raft sorting. Both Us9 and transferrin receptor are type II membrane proteins, and have 26 amino acids within their TMD. We constructed a PRV mutant that expressed a chimeric protein with the wild-type Us9 cytoplasmic domain, a transferrin receptor TMD, and wild-type 3 amino acid ectodomain (Figure 5A). This mutant, known as PRV 322, replicates with wild-type kinetics in porcine kidney (PK15) cells. Furthermore, the Us9-TfR protein is abundantly expressed in infected cell lysates and migrates more slowly by SDS-PAGE than wild-type Us9 (Figure 5C). Expression of the upstream and downstream genes, gE and Us2 respectively, were indistinguishable from Becker and suggested that recombination of Us9-TfR into the viral genome did not have polar effects on neighboring genes. Us9-TfR is efficiently incorporated into virions (Figure 5D), along with gE and Us2, but not UL34 which is a component of primary but not mature virus particles [65].

The localization and intracellular trafficking pattern of Us9 has been studied extensively in porcine kidney (PK15) cells [20],[26],[28]. To assess whether Us9-TfR remained a functional Us9 derivative (i.e. did not have an egregious trafficking defect), we examined the steady-state localization of Us9-TfR in transfected and infected PK15 cells. We hypothesized that both Us9 and Us9-TfR would have similar localization patterns as both contain the Us9 acidic domain, the region necessary for localization to a perinuclear cellular compartment [20]. Furthermore, sorting signals for the TfR protein reside in the cytoplasmic tail, not within the transmembrane domain [66]. Thus, the TfR TMD should be functionally inert in the context of Us9 trafficking.

**Figure 6:**
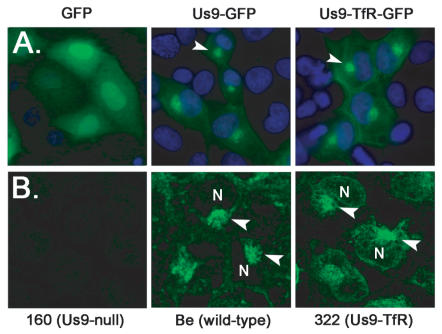
Localization of Us9-TfR in the absence and presence of infection. (A) PK15 cells were transfected with plasmids expressing GFP, Us9-GFP, and Us9-TfR-GFP. Cells were fixed with 4% paraformaldehyde at 24 hours post-transfection, and the nuclei stained with Hoechst 33342 (blue). Direct fluorescence was visualized using an inverted epifluorescence microscope using the appropriate excitation and emission filters. The arrowheads highlight the perinuclear, steady-state accumulation of Us9. (B) PK15 cells were infected with PRV 160 (Us9-null), Becker (wild-type), or PRV 322 (Us9-TfR) for 6 hours. Cells were fixed and stained with Us9 antiserum, and visualized on a Leica SP5 confocal microscope. Arrowheads denote Us9 accumulations adjacent to the cell nucleus (N).

The localization of Us9 fused to GFP mimics the localization of wild-type Us9 inside infected cells [20],[28]. We fused Us9-TfR to GFP to visualize its steady-state level in cells in the absence of infection. Both Us9-GFP and Us9-TfR-GFP were predominantly located to a perinuclear region in the cytoplasm of PK15 cells (Figure 6A). Confocal images of cells infected with Becker and PRV 322, fixed and stained with Us9 antiserum, also showed co-localization of Us9 and Us9-TfR to a perinuclear compartment, though the Us9-TfR signal was slightly more diffuse compared to the Us9 signal (Figure 6B). Overall, the trafficking of Us9-TfR inside transfected and infected cells was very similar to wild-type Us9.

**Figure 7:**
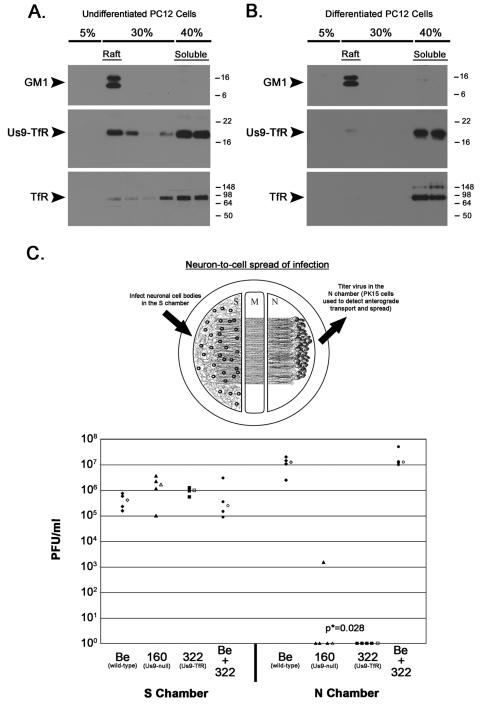
Raft association of Us9 is critical to anterograde spread of infection *in vitro.* (A) Non-differentiated and (B) differentiated PC12 Cells were infected with PRV 322 for 12 hours, and then lysed with cold 1% Triton X-100. Lysates were separated on a discontinuous Optiprep™ density gradient, and analyzed by SDS-PAGE. Western blot analysis was performed using biotinylated cholera toxin B subunit (for detection of GM1), and antiserum specific for Us9 and TfR. (C) Trichamber diagram illustrating the system used to measure PRV anterograde spread of infection from neurons to PK15 cells. SCG neurons were plated in the S chamber and allowed to extend neurites into the N chamber. The neurites were guided into the N compartment by a series of grooves. A monolayer of indicator PK15 cells were then plated on top of the axon termini in the N chamber. Cell bodies in the S chamber were infected, virus particles sorted into axons in a Us9-dependent manner, and these particles subsequently infected the PK15 cells that amplified the infection. Cultured neurons in the S chamber were infected at a high MOI with Becker (wild-type), PRV 160 (Us9-null), PRV 322 (Us9-TfR), or both Becker and PRV 322. Four chambers were used for each type of infection (closed symbols). At 24 hpi, medium and infected cells were harvested together from either the S or N chambers. Total plaque-forming units (PFU)/ml were determined for each chamber. The median value for the four samples is denoted by the offset open symbol. The *P* value (p*) was determined using the Wilcoxon two-sample test.

Next we tested whether the replacement of the wild-type Us9 TMD with that of transferrin receptor affected the affinity of Us9 for DRMs. We infected undifferentiated and differentiated PC12 cells for 12 hours, solubilized cells with 1% TX-100, and performed flotation analysis as done previously. Instead of Us9 being heavily enriched in the raft fraction as observed in Becker infected cells (see Figures 2 and 3), Us9-TfR was predominantly found in the soluble fraction, especially in differentiated PC12 cells (Figure 7A and 7B). To assess whether this impacted Us9 function in primary neurons, we took advantage of a trichamber neuronal culturing system [17],[37]. Dissociated SCG neurons are plated in the soma (S) chamber and allowed to mature for two weeks (Figure 7C). During this period, axons are directed between a series of grooves across the methocellulose (M) chamber to the neurite (N) chamber. A monolayer of indicator PK15 cells are then plated on top of the neurites in the N chamber. Cell bodies in the S chamber are infected, virus particles sort into axons in a Us9-dependent manner, and subsequently infect the PK15 cells that amplify the infection. The initial infection is confined to the S chamber via silicone vacuum grease and a methocellulose barrier. Therefore, infection spreads to the N chamber solely through axons that emanate from neuronal cell bodies and extend to PK15 cells [17]. We compared the anterograde transport and spread capabilities of PRV Becker (wild-type), PRV 160 (Us9-null), PRV 322 (Us9-TfR), and a co-infection of Becker and PRV 322 (Figure 7C, lower panel). Though all of the infections produced a comparable number of infectious virus in the S chamber, spread to second order PK15 cells in the N chamber was dramatically different. PRV Becker spread efficiently from neurons to PK15 cells, producing a median titer of 1.2×10^7^ PFU in the N chamber after 24 hours post-infection (Figure 7C). By contrast, the Us9-null mutant (PRV 160) did not spread to PK15 cells and no detectable infectious virus was produced in most dishes. However, in one dish, we detected a low number of infectious particles (1.5×10^3^). We interpret a low yield of amplified virus as a single neuron-to-cell spread event (the burst size of an infected PK15 cell is roughly 1000 PFU). Nevertheless, the neuron-to-cell spread capability of PRV 160 is extremely low compared to wild-type PRV Becker. PRV 322 (Us9-TfR) was completely defective in anterograde spread and was indistinguishable from the Us9-null mutant (no infectious virus detected in the N-compartment). This phenotype strongly correlated with the inability of Us9-TfR to target to lipid rafts/DRMs. When neurons were co-infected with both Becker and PRV 322, titers were virtually identical to those seen with Becker alone, indicating that PRV 322 does not have a *trans*-dominant effect on anterograde spread of infection.

**Figure 8:**
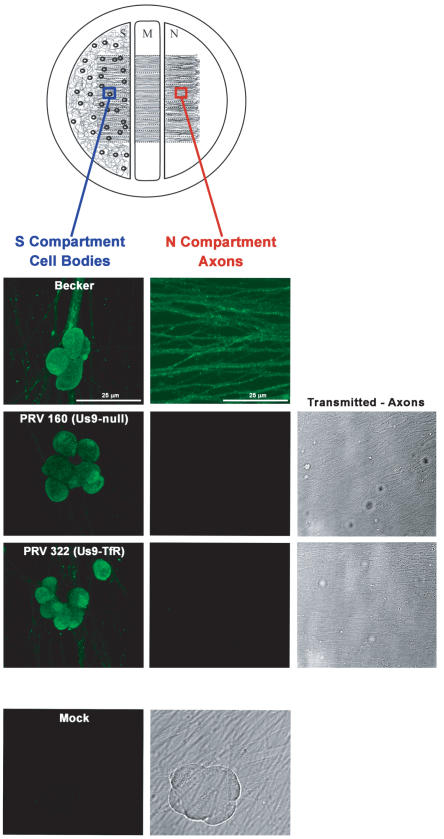
PRV 322 (Us9-TfR) is defective in axonal sorting of virus structural proteins. Trichamber diagram illustrating the system used to visualize PRV antigens in the cell bodies and axons of infected neurons. The blue box illustrates the site within the S compartment were cell bodies were imaged. The red box indicates the site were axons were imaged in the N compartment. SCG neurons were plated in the S chamber and allowed to extend neurites into the N chamber (through the M compartment). The neurites were guided into the N compartment by a series of grooves. Two weeks post-plating, cell bodies in the S chamber were infected at a high MOI with Becker (wild-type), PRV 160 (Us9-null) or PRV 322 (Us9-TfR). At 16 h postinfection, samples were fixed and labeled with PRV-specific polyclonal antiserum (Rb134) that recognizes virus glycoprotein and virus capsid proteins. All infected cell bodies within the S compartment stained for viral structural proteins (first column). Mock-infected cells did not label with the Rb134 antibody. Becker-infected axons stained heavily for PRV antigen (second column), though axons from PRV 160 and PRV 322 infected cell bodies were devoid of viral glycoprotein and capsid proteins (though an extensive network of axons was visible by transmitted brightfield illumination).

To assess whether the anterograde, neuron-to-cell spread defect for PRV 322 was at the level of axonal sorting of viral structural proteins (as previously shown for other Us9 mutants [15],[16]), we imaged infected neurons in the trichamber system using a PRV-specific antibody (made against acetone-fixed virus particles) that recognizes both virus glycoproteins and virus capsid proteins [21],[22]. PRV antigen was readily detected in the cell bodies of neurons in the S compartment infected with Becker, PRV 160, and PRV 322 (Figure 8, first column). Viral glycoprotein and capsid proteins were also abundant in the axons of Becker infected neurons within the N compartment (Figure 8, second column). By contrast, no viral structural proteins were observed by immunofluorescence in the axons of a Us9-null mutant (PRV 160) or the Us9-TfR strain (PRV 322), though an extensive network of axons was observed within the field of views by transmitted brightfield illumination (Figure 8, transmitted). These data suggest that the neuron-to-cell spread defect observed for PRV 322 (Figure 7) is the result of its inability to sort structural proteins into the axon of infected neurons.

Overall, these data are consistent with work done on the raft association of the influenza virus neuraminidase protein: substitution of a TMD domain that has a high affinity for lipid rafts, for one with a low affinity, dramatically alters DRM targeting and subsequent protein function.

## Discussion

We have demonstrated that Us9 is enriched in detergent-resistant membranes of non-polarized and polarized PC12 cells. This enrichment is cholesterol dependent, and is essential for Us9-mediated anterograde spread of infection in primary SCG neurons. Us9 is responsible for the axonal sorting of viral capsids [15], as well as the viral glycoproteins gB, gC, and gE [16]. These viral membrane proteins are associated with lipid rafts on the surface of PRV infected swine kidney cells and monocytes, and monospecific antibody-induced patching of one of these proteins led to the copatching of the others [18]. These patches were enriched in the raft marker GM1, but not transferrin receptor [18]. These findings are consistent with the raft partitioning of these viral glycoproteins in polarized PC12 cells (Figure 3). In addition, Us9-dependent targeting of viral membrane proteins requires maturation of cultured SCG neurons (Tomishima and Enquist, unpublished observations). Lipid raft formation during neuronal polarization is likely a key step in this sorting process [4]. Taken together, these data support a role for lipid rafts in the axonal sorting of alpha herpesvirus proteins and structures in the mammalian nervous system.

**Figure 9:**
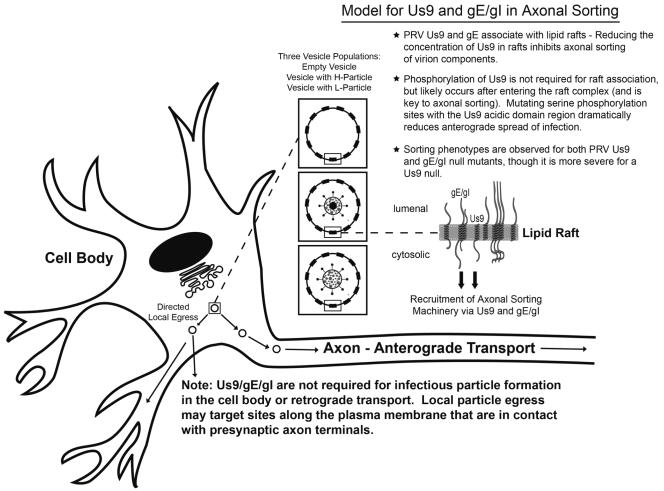
The role of Us9, gE/gI, and lipid rafts in anterograde sorting. Us9 and gE/gI enter the secretory pathway and associate with lipid rafts in the *trans*-Golgi network (TGN), the site of viral assembly. The presence of Us9 and gE/gI in lipid rafts, and subsequent Us9 phosphorylation, would recruit axonal sorting machinery to the cytoplasmic face of a small number of viral assembly complexes in the TGN, i.e. vesicles with viral membrane proteins only (empty vesicle), those containing mature virus particles (H-particle), or L-particles. A small number of assembly complexes would then bind a sorting adaptor protein(s) and go to axons; the majority of assembly complexes would target sites along the plasma membrane that are in contact with presynaptic axon terminals. Random egress is rare.

PRV gE, which is in a heterodimeric complex with the viral glycoprotein gI [22], is also necessary for the efficient axonal sorting of PRV structural components [54]. We propose a model in which Us9, gE/gI, and lipid rafts direct the sorting of vesicles into the axon of infected neurons (Figure 9). Us9 and gE/gI likely associate with lipid rafts in the *trans*-Golgi network (TGN), the putative site of viral assembly [20],[67],[68]. The presence of Us9 and gE/gI in lipid rafts (those decorating the surface of cellular vesicles) would recruit axonal sorting machinery to a small number of viral assembly complexes in the TGN, i.e. vesicles with viral membrane proteins only, those containing mature virus particles, or L-particles (illustrated as “three vesicle populations” in Figure 9). A limited number of vesicles containing virion components would then be targeted to the axon. Though a Us9-gE/gI complex in a lipid raft is required for efficient axonal transport, Us9 is clearly the more critical component, and the presence of gE/gI seems to enhance this process [17].

It is noteworthy that Us9/gE/gI are not required for infectious particle formation in the cell body of SCG neurons [16],[54], nor for retrograde transport in the mammalian nervous system [11],[69]. Furthermore, we have not observed the accumulation of viral capsids or membrane proteins in the cell body of a Us9-null, gE/gI-null, or Bartha strain lacking all three genes (unpublished observations). Our findings are consistent with a model in which the virus assembly and axonal sorting compartment within the TGN are identical (i.e. both processes use the same material for assembly). A small number of assembly complexes would bind a sorting adaptor protein(s) and go to axons; the majority of assembly complexes would egress the cell body locally, perhaps at sites where axons contact the infected cell body (Figure 9).

What determines whether a vesicle laden with viral structural proteins is directed to the axon as opposed to being released from the cell body? We propose that phosphorylation of Us9, subsequent to its recruitment into lipid rafts, may be the pivotal step. The PRV Us9 acidic domain region is heavily phosphorylated during infection [26], and is essential for anterograde, transneuronal spread *in vivo* [26]. Phosphorylation occurs predominantly on two serine residues within the 10-amino-acid acidic domain, and mutating these serines to alanines dramatically decreases anterograde spread in the rat visual system [26]. This phosphorylation event would occur after Us9 enters the raft, as the acidic cluster domain is not required for raft association of Us9 (Figure 4B). We are currently investigating whether 1) the association of Us9 with lipid rafts coincides with its phosphorylation inside infected cells and 2) the phosphorylation state of the Us9-TfR chimera is reduced since it is no longer enriched in lipid rafts.

Perhaps not surprisingly, the Us9 acidic domain region is highly conserved among Us9 homologs of other neurotropic herpesviruses, including the human pathogens herpes simplex virus (HSV) and varicella zoster virus (VZV), as well as the animal pathogens equine herpesvirus (EHV) and bovine herpesvirus (BHV), suggesting an important role for this domain in the anterograde spread of virus in the mammalian nervous system [26], [70]–[72]. We predict that phosphorylation of the Us9 acidic domain (within the context of a lipid raft) is necessary for binding an axonal sorting adaptor, which would then mediate anterograde transport inside the axon [16],[73].

Our model addresses how viral glycoproteins/vesicles are sorted into the axon of infected neurons, but does not suggest a mechanism for their function in cell-to-cell spread of infection in cultured epithelial cells [74]–[76]. It may be that these two processes are fundamentally different since 1) gE mutants with a small-plaque phenotype on MDBK cells have wild-type anterograde spread kinetics in the rat visual system [77], 2) deletion of PRV Us9 has no effect on cell-to-cell spread of infection in epithelial cells, but a dramatic impact on anterograde sorting [11], and 3) gB mutants with a small-plaque phenotype on ST cells [78] have wild-type anterograde neuron-to-cell spread kinetics in our trichamber system (Curanovic and Enquist, unpublished findings).

It was intriguing to discover that PRV gH was not associated with detergent-resistant membranes in non-polarized and polarized PC12 cells, and was completely solubilized with 1% TX-100 (as was the non-raft marker transferrin receptor). The virus fusion machinery is composed of gB trimers, as well as gH/gL heterodimeric complexes (reviewed in [79]). PRV gH is essential for entry into uninfected cells, cell-to-cell spread of infection in tissue culture [80], and transneuronal spread of infection in mice [81]. Does gH enter the axon in a Us9 or gE-dependent manner despite its apparent exclusion from DRMs? We are currently investigating this question. Cross-linking experiments performed on purified HSV virions found that hetero-oligomers of gB, gC, and gD were closely associated with one another in the virion envelope (within 11.4Å). The gH and gL proteins could also be cross-linked within the envelope as one might predict. Interestingly, gL was never cross-linked to gB, leading the authors to suggest that organization of these proteins in the membrane “precludes associations of gH/gL with gB” [82]. One explanation for this finding is that gB is present in a lipid raft microdomain, whereas gH is not (a small proportion of HSV gH has been shown to be in the DRM fraction of infected COS cells [83]). It is also feasible that PRV gH may indeed be raft-associated, but solubilization with cold detergent is too stringent. Triton X-100 and CHAPS are reported to be the most reliable detergents for analyzing raft association [84]. However, some membrane proteins solubilized by Triton X-100 do associate with lipid rafts by antibody copatching [18],[51],[85],[86]. At this time it is unclear if gH has a weak affinity for rafts, or is indeed a “true” non-raft protein as is transferrin receptor.

Our findings also highlight the importance of the Us9 TMD domain in raft targeting. Several studies have addressed the importance of the transmembrane segment in partitioning viral and cellular membrane proteins into lipid rafts: influenza virus hemagglutinin [60],[64] and neuraminidase [59],[63], the LMP-1 oncoprotein of Epstein-Barr virus (EBV) [87], and the human immunoreceptor FcγRIIA [88]. It is clear from these studies that amino acids within the TMD (even single amino acids) have dramatic effects on raft partitioning, sorting, or signaling events. A comprehensive analysis of the Us9 TMD may reveal residues important for protein-lipid/protein-protein interactions that are key in promoting axonal sorting of mature virus particles.

Several alpha herpesvirus proteins have been shown to associate with DRMs during virus replication. In addition to PRV membrane proteins ([18]; this study) the virion host shutoff (vhs) protein of HSV-1 was shown to be enriched in organellar membrane fractions which contain virus assembly intermediates [83]. HSV gB is proposed to mobilize lipid rafts during entry, perhaps to mediate cell signaling [81]. The UL11 protein of HSV-2, a myristoyl and palmitoyl tegument protein, associated with DRMs of infected Caco-2 cells [89]. The UL56 protein of HSV-2, a tail-anchored type II membrane similar to PRV Us9, was present in detergent-insoluble lipid rafts; it is predicted to be involved in vesicular trafficking in HSV-2 infected cells [90]. These findings demonstrate that lipid rafts play an important role in the replication cycle of alpha herpesviruses, and underscore the importance of lipid rafts in virus biology [91]–[94].

Two competing models have been presented for anterograde, axonal transport of alpha herpesviruses in neurons (reviewed in [95]). Viral capsids are either transported down the axon independently from viral membrane proteins (and assemble prior to egress), or they are sorted and transported together as a mature virus particle within a vesicle. Recent studies have shown that efficient capsid transport is dependent on at least two viral membrane proteins, Us9 and gE. Deleting either of these genes from HSV or PRV results in a marked decrease of viral capsids from entering axons of infected neurons [12],[15],[54],[96]. It is difficult to conceive how viral membrane proteins could impact capsid sorting unless the two were tightly coupled during axonal entry and transport (i.e. if capsids entered axons separate from viral membrane proteins, deletion of gE and Us9 would have no effect on capsid sorting). These recent findings are consistent with a model where viral capsids and membrane proteins traffic together in axons as mature virus particles (within a vesicle) (Figure 9).

In conclusion, we have shown that PRV Us9 is highly enriched in DRMs of non-polarized and polarized PC12 cells, and this enrichment is critical to axonal targeting and subsequently in neuron-to-cell spread. This is the first report to implicate lipid rafts in the axonal sorting of alpha herpesvirus structural proteins in mammalian neurons. Our future plans include isolating lipid rafts from polarized PC12 cells infected with wild-type PRV, and identifying the cellular and viral proteins present within these lipid microdomains.

## Supporting Information

Video S1 A differentiated PC12 cell body (with multiple neurites) infected with PRV GS443 for 12 hours. Green capsid puncta are readily observed moving in the anterograde direction, i.e. away from the cell body, in all visible neurites. The cell body was imaged for approximately 13.5 minutes. Each frame is a 2D projection representing a stack of approximately 15 optical sections, 0.5 µm apart (6.36 seconds/frame). The playback rate is 7 frames/sec.
(5.46 MB MOV)

Video S2 A differentiated PC12 cell body infected with PRV 368 for 12 hours. No capsid puncta were observed moving in the anterograde direction in neurites over a 12.5 minute period. Each frame is a 2D projection representing a stack of approximately 15 optical sections, 0.5 µm apart (6.88 seconds/frame). The playback rate is 7 frames/sec.
(4.36 MB MOV)

Video S3 A differentiated PC12 cell body infected with PRV 368 for 12 hours. No capsid puncta were observed moving in the anterograde direction in neurites over a 15 minute period (capsids were not present beyond the proximal segment). However, several capsids are undergoing transneuronal, retrograde transport from the infected cell body to an uninfected cell above the field of view (see Movie S4). Despite an abundance of moving capsid puncta within the cell body, no other egress events are visible. Each frame is a 2D projection representing a stack of approximately 15 optical sections, 0.5 µm apart (6.98 seconds/frame). The playback rate is 7 frames/sec.
(10.50 MB MOV)

Video S4 Capsids from the same infected cell body in movie S3 are transported in a retrograde manner to an uninfected cell above. A capsid is shown to traffic back to the uninfected PC12 cell body, enter the cell, and move to a perinuclear region where capsids are accumulating, perhaps the microtubule organizing center (MTOC). Note that no capsids are moving in the anterograde direction inside the infected cell. Each frame is a 2D projection representing a stack of approximately 15 optical sections, 0.5 µm apart (6.7 seconds/frame). The playback rate is 7 frames/sec.
(11.13 MB MOV)
